# Bacteria with Phosphate Solubilizing Capacity Alter Mycorrhizal Fungal Growth Both Inside and Outside the Root and in the Presence of Native Microbial Communities

**DOI:** 10.1371/journal.pone.0154438

**Published:** 2016-06-02

**Authors:** Yuli Marcela Ordoñez, Belen Rocio Fernandez, Lidia Susana Lara, Alia Rodriguez, Daniel Uribe-Vélez, Ian R. Sanders

**Affiliations:** 1 Biology Department, Faculty of Science, Universidad Nacional de Colombia, Ciudad Universitaria—Avenida Carrera 30 N° 45–03, Bogotá, Colombia; 2 Faculty of Agronomy, Universidad Nacional de Colombia, Ciudad Universitaria—Avenida Carrera 30 N° 45–03, Bogotá, Colombia; 3 Biotechnology Institute, Universidad Nacional de Colombia, Ciudad Universitaria—Avenida Carrera 30 N° 45–03, Bogotá, Colombia; 4 Department of Ecology and Evolution, Biophore Building, University of Lausanne, 1015, Lausanne, Switzerland; Estación Experimental del Zaidín (CSIC), SPAIN

## Abstract

Arbuscular mycorrhizal fungi (AMF) and phosphate solubilizing *Pseudomonas* bacteria (PSB) could potentially interact synergistically because PSB solubilize phosphate into a form that AMF can absorb and transport to the plant. However, very little is known about the interactions between these two groups of microorganisms and how they influence the growth of each other. We tested whether different strains of bacteria, that have the capacity to solubilize phosphate, are able to grow along AMF hyphae and differentially influence the growth of AMF both outside the roots of carrot in *in vitro* conditions and inside the roots of potato in the presence of a microbial community. We found strong effects of AMF on the growth of the different bacterial strains. Different bacterial strains also had very strong effects on the growth of AMF extraradical hyphae outside the roots of carrot and on colonization of potato roots by AMF. The differential effects on colonization occurred in the presence of a microbial community. Our results show that these two important groups of rhizosphere microorganisms indeed interact with each other. Such interactions could potentially lead to synergistic effects between the two groups but this could depend on whether the bacteria truly solubilize phosphate in the rhizosphere in the presence of microbial communities.

## Introduction

Arbuscular mycorrhizal fungi (AMF) are present in nearly all soils, forming associations with roots of approximately 80% of plant species [1]. In exchange for photosynthates, AMF provide phosphate (P) and other nutrients to their plant hosts by producing hyphae that grow out from roots, effectively increasing the soil volume from which minerals are acquired [1]. The capacity of AMF to transport P to the plant, which in some cases adds up to 70% of total P plant uptake, is well known [1]. However, AMF can only exploit soluble P sources and much P in the soil is in an insoluble form.

The rhizosphere is regarded as a hotspot for microbial activity and recent studies indicate that this is also the case for the mycorrhizosphere where soil bacteria may attach to extraradical AMF hyphae [2]. The effect of arbuscular mycorrhizal fungal colonisation on the soil microbial community has been little studied. Changes in soil bacterial community composition due to the presence of AMF have been described, both *in vivo* and *in vitro* [3, 4]. Among soil bacteria in the rhizosphere, those with the capacity to solubilize P are highly relevant when studying AMF interactions because these bacteria could potentially make available more soluble P for absorption by AMF hyphae.

Phosphate solubilizing bacteria (PSB) are free-living soil microorganisms that are present in most soils [5]. In *in vitro* conditions, they have the potential to improve availability of P to the plant by solubilizing organic and inorganic P, through the action of synthetized phosphatases, by lowering the pH of the soil, and/or chelating P from Al^3+^, Fe^3+^, Fe^2+^ and Ca^2+^ with the help of organic acids [5, 6]. The most widely used method to initially select microorganisms with P solubilization capacity *in vitro* is the use of tri-calcium phosphate, although the predictability of this assay for P solubilization in soil conditions is very limited (recently reviewed in [7]).

Many soil bacteria, including species of *Pseudomonas*, *Azotobacter*, *Burkholderia*, *Bacillus* and *Rhizobium* have been shown to have the capacity to solubilize poorly available P [6, 8, 9]. In particular, *Pseudomonas spp*., are known to colonize the rhizosphere, solubilize P and can exhibit additional plant growth promoting characteristics such as plant growth stimulation and the production of metabolites that have anti-microbial activity [10, 11].

However, the interactions between PSBs and AMF are poorly understood and the approach to using both these microbial groups for applications in agriculture is often naive because of variation in soil abiotic and biotic environments in which these organisms have often not been tested [12, 13]. Moreover, very often, only single strains of these microbial groups have been shown in laboratory or greenhouse conditions to have the capacity to solubilize P or to improve plant P acquisition. Indeed, higher plant P uptake capacity has previously been reported when plants are co-inoculated in greenhouse conditions, with AMF and PSB [14, 15]. These bacteria probably improve the availability of P, which can subsequently be efficiently absorbed by AMF hyphae [14, 16]. Thus, on the basis of results, mostly from artificial experiments conducted in sterilized soil, AMF and PSB are thought to act synergistically. Recent evidence also points, not only to synergistic effects between AMF and PSB but also to cooperation between these organisms [17]. However, most of the beneficial effects of AMF are observed in experiments conducted in sterile soil [13]. In reality, plants naturally become colonized by the local AMF community. A more realistic test of their potential is whether adding AMF inoculum and PSBs to unsterilized soil will give a growth benefit to the plant. Such tests are rarely performed. Isolated beneficial microbes are then used in field applications, where the bacteria and fungi encounter both diverse soil environments and diverse microbial communities, including existing diverse populations of both PSBs and AMF. It is perhaps unsurprising; therefore, that application of both AMF and PSBs in agriculture has had very variable success [12].

Given that both AMF and PSB must have co-existed in the rhizosphere for millions of years, many possible interactions could have evolved between them. Yet the interaction between AMF and PSB is not well understood. Firstly, in the mycorrhizosphere, the soil zone influenced by both the roots and the mycorrhizal fungi [18], AMF exudates create an environment that can influence bacterial growth [2, 4, 19]. Attachment of bacteria, with P solubilizing capacity, to extraradical AMF hyphae, could ensure that P solubilizing activities of the bacteria would be located in the zone where they can be most beneficial in allowing the fungi access to additional soluble P. At the same time, attachment to the AMF hyphae might provide bacteria with a route to efficiently access the mycorrhizosphere [20]. Some soil bacteria have been shown to attach both to vital and non-vital AMF hyphae in *in vitro* conditions [2]. However, none of the bacteria in that study were assessed for their P solubilizing capacity. It is unknown whether any bacteria with phosphate solubilizing capacity have the ability to attach to AMF extraradical hyphae [21]. Of those PSB that might associate with AMF hyphae, it is unknown whether these bacteria might influence either the growth of AMF inside the roots or of AMF hyphae outside the roots. A positive effect of PSB on extraradical AMF hyphal growth could help PSB to access new areas of the mycorhizosphere and increase access by AMF hyphae to new sources of solubilized P. Thirdly, populations of PSB are diverse in the soil [6, 22–25] and it is unknown whether there is variation among strains in the effects of PSB on AMF.

The aims of this study were to test: 1. To test whether different *Pseudomonas* spp strains, which have previously been shown to be capable of solubilizing P in *in vitro* conditions, differ in their ability to grow along AMF hyphae; 2. Whether the bacterial strains differentially influence the growth of the fungus outside the roots and in the absence of a microbial community and whether P solubilized by the bacteria can be transported by the fungus to the plant root; 3. Whether there is variation in the capacity of the bacterial strains to influence colonization by mycorrhizal fungi inside the roots and in the presence of a microbial community in a non-sterilized Colombian Andisols. We used ten *Pseudomonas* spp. strains, from Colombian Andisols that were previously isolated [26] and that were characterized as PSBs in this study. Here we define PSB as bacteria that have been shown to solubilize tri-calcium phosphate in *in vitro* conditions. Thus, the bacteria we consider possess the metabolic capability to solubilize phosphate. This, however, does not mean that these bacteria would indeed solubilize P in a variety of different soils and in the presence of a potentially competing microbial community. We focussed on PSB originating from Colombian Andisols because these soils are important for potato production but characterized by very high P retention due to acidic conditions (pH <5.5). We used the *in vitro-*produced AMF *Rhizophagus irregularis* because it has been shown to improve plant growth in field conditions in non-sterilized tropical acidic soils and has a global distribution [27].

## Materials and Methods

### Microbial inocula

The AMF *Rhizophagus irregularis* (isolate DAOM 197198) was grown on *Agrobacterium rhizogenes*-transformed carrot roots, which were established and maintained in minimal medium (MM) for 90 days at 25°C [28].

Ten strains of *Pseudomonas sp*. were previously isolated from the potato rhizosphere at different altitudes in the Colombian Andes [26]. They were characterised according to their P solubilizing capacity *in vitro*, using two different methods. Phosphate solubilizing capacity of the bacteria was first carried out following the molibdovanadate method by using the Spectroquant^®^ kit (Merk Millipore Corporation) (S1 Table). Second, the bacterial capacity to solubilize aluminium phosphate was measured, according to Nautiyal [29] (S1 Table). The bacteria were also assessed for their ability to produce indol acetic acid and indol-related substances. The production of indol-related substances was determined b y growing the PSB in LB medium with 0.3 mM of L-tryptophane following the procedure of Glickmann and Dessaux [30] (S1 Table).

### Characterisation of the bacterial strains

By the use of the multilocus sequence analysis (MLSA), the most accepted method for phylogenetic assignation of *Pseudomonas* strains [31], the taxonomic affiliation of the bacteria used in this work was established. We sequenced four amplicons of constitutive genes per strain; namely *rpoD* and *gyrB* [32], pyrroloquinoline quinone (*pqqC*) [25] and 16S rDNA [33] for independent analysis by sequence similitude.

The conditions specified in [32] were followed for the genes *rpoD* and *gyrB*. Briefly, polymerase chain reaction (25 μl) contained 1 ng of bacterial DNA in 3 μl, 1 x PCR buffer (Thermo Scientific), 5% bovine serum albumin (10 g l-1, Thermo Scientific), 100 mM each of dATP, dCTP, dGTP and dTTP, 0.40 mM of each primer and 1.0 U of Taq DNA polymerase (Thermo Scientific). The initial denaturation at 94°C (150 s) was followed by 30 PCR cycles 94°C for 30 s, 65°C for *gyrB* and 60°C for *rpoD* for 30 s and 72°C for 60 s and a final extension at 72°C (10 min). PCR amplification of *pqqC* from bacterial DNA was carried out following [25], with some modifications. In a final volume of 25 μl, the PCR mix contained 100 μM of each dNTP, 0.4 μM (each) forward and reverse primer, 0.75 U *Taq* DNA polymerase (Thermo Scientific), 5% bovine serum albumin, and 3 μl of genomic DNA. The following thermocycling conditions were used: initial denaturation at 96°C for 10 min followed by 30 cycles of 96°C for 30 s, 65°C for 30 s, 72°C for 1 min, and a final elongation at 72°C for 10 min. The procedure for the 16S rRNA gene amplification followed the method described in [33].

Sequences from each bacterial strain were first compared using the BLAST procedure to sequences in the NCBI database. They were also compared using BLAST to sequences in PseudoMLSA; a more specialized sequence database for *Pseudomonas* spp. [31]. Sequences from the two housekeeping genes gyrB and rpoD were also aligned independently with the program CLUSTAL_X [34]. The sequences were concatenated, giving a fragment of 1285 bp, for final alignment. A maximum likelihood tree was inferred using the MEGA program, version 6.0 [35]. The heuristic algorithm Nearest-Neighbor-Interchange was used and all positions containing gaps and missing data were eliminated from the dataset (Complete Deletion option). The general-time-reversible method with estimated gamma correction was chosen as the substitution model. A bootstrap analysis of 1000 replications was also performed. The topologies of the trees were visualized using the FigTree program, version 1.4.2 (http://tree.bio.ed.ac.uk/software/figtree/).

The bacterial strains were also compared for their ability to swim and swarm as these are thought to be traits associated with the ability to form biofilms [36] (S2 Table). The following modifications to the protocol by [36] were made: No NH_4_Cl was added to the medium used to measure swarming but the medium was supplemented with 0.2% glucose and 0.05% glutamate; and to measure swimming, 0.3% LB medium was used. For swarming and swimming assays, fresh bacterial colonies were suspended in sterilized saline solution 0.85% (w/v) and adjusted to a concentration to 1 x 10^7^ ml^-1^ colony forming units (cfu) for the assays. Saline solution was used as a blank. Adherence of the different PSB strains to abiotic surfaces was determined [37] (data in S2 Table). No detectable values of the three variables were recorded in the blank treatments.

For the experiments 1, 2 and 3 described below, the bacterial strains were grown in LB agar for 48 h and re-suspended in saline solution 0.85% (w/v) to the desired concentration. All bacterial strains used were rifampicin-resistant mutants.

### Experiment 1: To study the growth of PSB strains on the surface of extraradical mycorrhizal fungal hyphae and measure fungal growth

Dual-compartment cultures of AMF were established in split 90 x 15mm Petri dishes [38]. The proximal (root) compartment was filled with 20 ml of a minimal growth medium (MM) [39]. The distal (hyphal) compartment contained 8 mL of MM without sucrose or phosphorus, creating a slope from the bottom of the dish to the top of the plastic separation [4]. This slope was made to facilitate the growth of AMF extraradical hyphae from the root to the distal compartment that lacked roots. The pH of each medium was adjusted to 5.5 and Phytagel® 3.5 g l^-1^ was added as gelling agent before sterilization at 121°C for 15 min.

To establish dual-compartment AMF cultures, a transformed carrot root fragment (approximately 5cm length) was put onto the medium in the proximal compartment of each plate and inoculated with AMF by transferring the medium (in blocks of approximately 1 cm^2^) containing spores and extraradical mycelium that came from the proximal compartment of another culture. Cultures were examined weekly and roots were trimmed aseptically to prevent their growth into the distal compartment. Thirty-three dual-compartment AMF cultures, with well growing extraradical hyphal growth in the distal compartment, were set up in this way.

After 48 days, 12 ml semi-liquid MM (Phytagel 1g l^-1^) without sucrose or P was poured onto the MM medium in the distal compartment and four equidistant wells were made (2 cm apart from each other and 0.5 cm from the Petri dish periphery). Twenty-five μl of sterilized saline suspension of a PSB strain (1 x 10^8^ cfu ml^-1^. concentration) was then placed in each well. In the control treatment, the plates were inoculated with the same solution that did not contain bacteria and were checked for absence of bacterial growth at the end of the experiment. Each PSB treatment and the control was replicated three times, giving a total of thirty-three dual-compartment cultures. Petri plates were incubated horizontally at 25 +/-1°C in the dark for 30 days, after which hyphal growth and bacterial growth was measured, in the distal compartment, under a stereo microscope. Controls were checked for the absence of bacteria in the hyphal compartment and on the surface of AMF hyphae but the length of extraradical hyphae in the control treatments was not measured. Extraradical hyphal length was quantified in the distal compartment following a gridline intersect method [40] in 25 randomly selected squares, each measuring 0.55 cm^2^. The 25 values per plate were then summed and divided by 25 to give one hyphal length value per replicate plate. In the same 25 randomly selected 0.55 cm^2^ squares, measurements were made of the length of bacterial colonies that developed along AMF extraradical hyphae by recording at each intersection whether bacterial colonies were present on the hyphae. The 25 values of each measurement were then summed and divided by 25 to give one value per replicate of each treatment for further statistical analysis. In addition, observations were also made on each plate in the root compartment to score whether the bacteria adhering to the AMF hyphae had also moved from the distal to the root compartment, and were growing on or adjacent to the roots and on the medium. Spores were also quantified in 25 randomly selected squares of 1 cm^2^ area.

### Experiment 2: *In vitro* assay to assess P acquisition by roots in AMF cultures inoculated with different PSB strains

Dual-compartment AMF cultures were established as described above, except that the distal compartment, contained semi-liquid MM (Phytagel 1g l^-1^) without sucrose or soluble P. The medium in the distal compartment also contained a suspension of insoluble tri-calcium phosphate (5g l^-1^). The same ten bacterial strains were added as described above, in the same concentration in the distal compartment. Three replicates were established for each treatment. Dual additional treatments were set up with the same number of replicates. These were a control in which sterilized saline solution, but containing no bacteria, was added to the distal compartment (subsequently referred to as control) and a treatment with no bacteria and no tri-calcium phosphate in the distal compartment (subsequently referred to as NTP). After the addition of the bacterial inoculum or solution without bacteria, the Petri dishes were incubated at 25 ±1°C in the dark. After 20 days, the roots were harvested, dried and ground. The phosphorus concentration in transformed carrot roots was measured by the molybdate blue method [41].

### Experiment 3: Greenhouse assays to assess combined PSB and AMF effects on plant growth and PSB effects on AMF colonization of roots

Tuber seeds of *Solanum tuberosum* group phureja [42], were sterilized with ethanol (75% v/v) and sodium hypochlorite (1% v/v), and washed with sterile distilled water. The same ten bacterial strains that were used in Experiments 1 and 2 were used to inoculate seed tubers. Several seed tubers were immersed in 15 ml of a bacterial suspension (containing 1 x 10^8^ colony forming units ml^-1^) of each one of the PSB strains, for 15 minutes with agitation every three minutes. After this time, three seed tubers from each of the ten bacterial treatments were randomly selected in order to measure the number of recoverable rifampicin-resistant mutant (RRM) bacteria. Briefly, one seed tuber was immersed in 10 ml of sterilized saline solution (SSS), mixed by vortex for 1 min and serial 10-fold dilutions were made by dilution with the same saline solution. RRM concentration per seed tuber was determined by plating 100 μl aliquots of the serial dilution onto LB with rifampicin (100 μg ml^-1^) agar. Following 48 h of incubation at 28°C, bacterial colonies were counted (CFU per seed tuber). Tuber seeds immersed in SSS were used as control. Thus, the numbers of recoverable rifampicin-resistant mutant bacteria from the surface of the seed tubers in each treatment at the beginning of the experiment was measured.

Non-sterilized soil from a privately owned agricultural field in the Colombian Andes (in the Madrid municipality in the Department of Cundinamarca, Colombia), that had been under potato cultivation, was used for the experiment. The owner of the land gave permission to use the soil from the site for this study. No other specific permissions were required for the use of the soil and the study used no endangered species or protected species. The soil had a pH of 6.3 and contained 34 g kg^-1^ of extractable phosphate (see S3 Table for a detailed chemical analysis of the soil). Rock phosphate was mixed with the soil so that the final P concentration of the mixture contained an equivalent of 150 kg P ha^-1^ of additional P. The source of rock phosphate was Granufos 20® which contains 20% phosphate as P_2_O_5_. One seed tuber that had been immersed in the suspension of one of the ten bacterial strains was sown in each pot. One ml of a suspension containing 2000 propagules of the AMF *Rhizophagus irregularis* was dispersed onto the surface of the seed tuber that was then covered with soil. Thus, there were ten treatments where the seed tubers had been immersed in a bacterial suspension. Four additional treatments were made: 1. Plants were inoculated with *Rhizophagus irregularis* but the seed tubers had not been immersed in a bacterial suspension (subsequently called AMF + insol P). 2. The soil and rock phosphate mix was used but the seed tubers had not been immersed in a suspension of bacteria and no AMF inoculum was added (subsequently called Insol. P). 3. Soil was added to the pot but was given a solution of K_2_HPO_4_, which is a soluble form of P, (subsequently called Sol. P) and 4. Pots contained only the soil, with no added P or microbes. However, this treatment and all other treatments contained bacteria and fungi that naturally occurred in the soil, as it was unsterilized. There were four replicates of each of the 14 treatments. All seed tubers seed had sprouted at the time of inoculation and sowing. Pots were filled with a 2.8 kg mix of rock phosphate and non-sterilized soil from a field in the Andes (1:4), which had been under potato cultivation.

Pots were maintained in a growth room with a photoperiod of 16/8 h light/dark, 16°C, and 60% RH, and were watered three times per week to field capacity. Position of the pots was randomized in the growth room. At the end of experiment, the numbers of recoverable rifampicin-resistant mutant bacteria from the soil was measured in each treatment. Rhizospheric soil samples were taken by carefully removing potato plants from the soil, from each pot. Plants were removed and shaken to discard excess soil, and roots were separated from shoots. Soil tightly adhered to roots was kept. One gram of rhizospheric soil, per plant, was diluted in 9 ml of sterilized saline solution 0.85% (w/v), mixed by vortex for 1 min and serial 10-fold dilutions were done. RRM concentration (CFU per gram of rhizospheric soil) was determined by plating 100 μl aliquots of the serial dilution onto LB with rifampicin (100 μg ml^-1^) agar. Following 48 h incubation at 28°C, bacterial colonies were counted. Mycorrhizal colonization of the roots was evaluated 70 days after sowing [43]. Briefly, 30 randomly chosen 1 cm-long pieces were cut from each root system, cleared for 10 min at 60°C in 10% KOH, washed with distilled water for three times, and submerged in HCl 10% for 10 min, stained with trypan blue in lactic acid overnight and mounted on a slide. Histochemical staining was also used to evaluate alkaline phosphatase activity in mycorrhizal roots [44]. Mycorrhizal colonization was evaluated microscopically. All plants per treatment were collected and dried in order to determine the root and shoot dry weight and leaf phosphorus concentration. Phosphorus concentration was measured as above.

### Statistical analysis

In the first experiment, we used a one-way analysis of variance (ANOVA) with 10 levels to assess whether the different PSB isolates had a significant effect on the growth of extraradical AMF hyphae, AMF sporulation and whether the different PSB grew differently on the surface of AMF extraradical hyphae.

In the second experiment, we performed a one-way ANOVA with 12 levels to assess whether there were significant differences in *in vitro* root P concentration among plates inoculated with different PSB strains.

In the 3^rd^ experiment, the number of bacteria in the rhizosphere, AMF colonization of the roots, metabolically active AMF colonization, leaf P concentration and the root and shoot dry weights, total dry weight were each subjected separately to one-way ANOVA with 14 levels. Means comparisons following ANOVA in all experiments were made with a Tukey test, except that a Dunnet’s test was used for the amount of Tricalcium phosphate solubilized in vitro and for the production of IAA and indol-related substances. In addition, Pearson’s correlation coefficient was used to examine whether correlations existed between pairs of the variables measured in the greenhouse experiment.

## Results

### Characterization of strains and phylogenetic analysis

The ten strains of bacteria were all demonstrated to have P solubilizing capabilities *in vitro* using 2 different methods (S1 Table). Strain P63 and P95 had the highest capacity to solubilize phosphate *in vitro* while strain P80 had the lowest capacity. The strains also varied significantly in their production of indol-related substances, with stain P80 exhibiting a higher production of indol-related substances than strains P28, P29, P36, P95 and P102 (S1 Table). The strains also differed in their ability to swim and swarm (S2 Table). Strains P29, P63, P80 P102 and P104 swam faster than P36, P95 and P108. Swarming ability was significantly highest in strains P80 and P95. The strains also varied greatly in their ability to attach to abiotic surfaces (S2 Table). Strains P36 and P63 were able to adhere to abiotic surfaces most successfully and significantly more than all other strains. P29 showed the lowest ability to adhere to abiotic surfaces.

Sequences of the four genes from the ten bacterial strains were obtained, except for the *pqqC* gene in strain 95, which was poor quality (S4 Table). The results of both BLAST analyses (S4 Table) and phylogenetic analysis (S1 Fig) suggested that the 10 bacterial strains were affiliated with the *Pseudomonas fluorescens* lineage. There was no match with species of the *P*. *aeruginosa* lineage. Sequences were deposited in the GenBank database: KU048076-KU048095 accession numbers.

### Experiment 1

**Fig 1:**
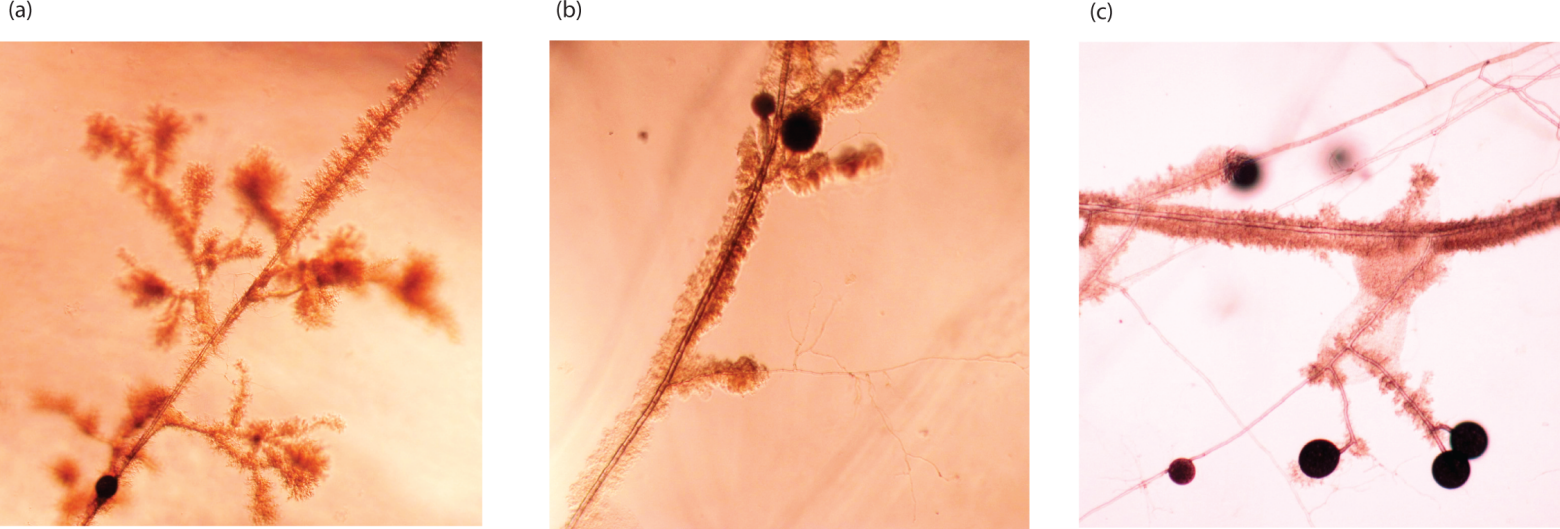
Photographs of bacterial colonies on the hyphae of *R*. *irregularis* in *in vitro* cultures in experiment 1. a. Growth around branching absorbing structures; b. on and around hyphae; c. around subtending hyphae of *R*. *irregularis*.

**Fig 2:**
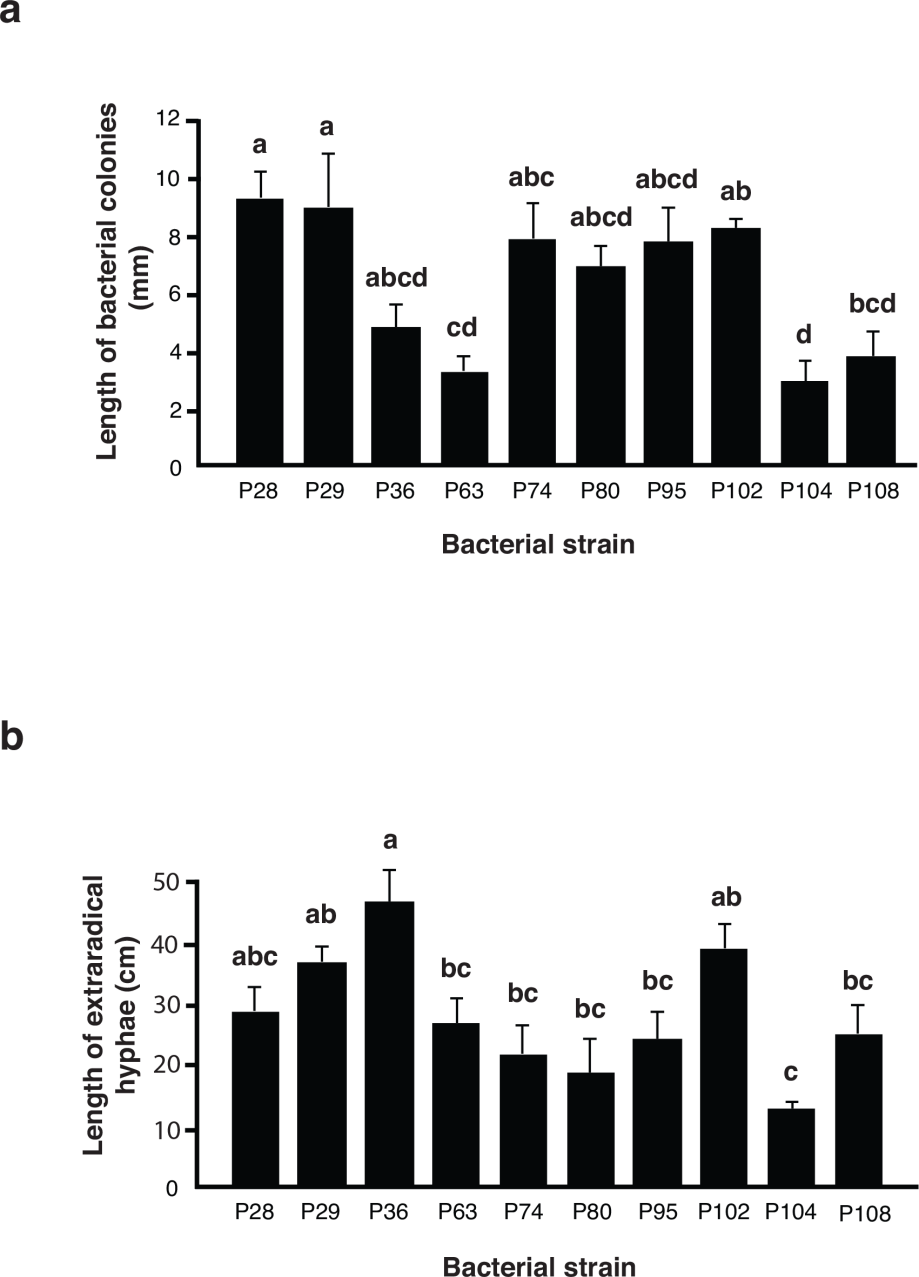
Growth of bacteria and growth of extraradical hyphae in Experiment 1. a. Growth of ten different P solubilizing bacteria strains on the surface of extraradical hyphae of *R*. *irregularis* growing in the distal compartment of a split *in vitro* culture in experiment 1. b. Growth of extraradical hyphae of *R*. *irregularis* in the distal compartment of a split *in vitro* culture in the presence of ten different P solubilizing bacteria strains. Measurements represent mean length per treatment per 1 cm^2^. Error bars represent + 1 S.E. Different letters above bars represent significant differences (*P* ≤ 0.05) according to a Tukey test.

In experiment 1, all bacterial strains were able to grow on the culture medium in the distal compartment in the presence of AMF hyphae. Microscopic observations confirmed that bacteria attached to and grew along the AMF hyphae in the distal compartment, often forming thick colonies around thick hyphae as well as around subtending hyphae on which spores had formed (Fig 1A–1C). Extraradical hyphae of AMF are not normally very branched except when they form so-called branching absorbing structures (BAS) [45]. The PSB strains P74, P80, P95 and P102 all formed bacterial colonies all around the hyphae of BAS (Fig 1A). All ten bacterial strains grew along the extraradical hyphae of *R*. *irregularis* but the length of AMF hyphae colonized by the bacteria differed significantly among PSB strains (ANOVA F _(9, 20)_ = 6.23, *P* = 0.0003; Fig 2A). Strains P28, P29 and P102 grew significantly more along AMF extraradical hyphae than strains P63 and P104. We did not observe that PSBs accessed the carrot roots by growing along the AMF extra radicle hyphae and onto and around the carrot roots. There was no bacterial growth in the control treatment. The growth of AMF, as measured by AMF extraradical hyphal length, was differentially affected by the presence of different PSB (ANOVA F _(9, 20)_ = 6.21, *P* = 0.0003; Fig 2B). The highest extraradical hyphal growth was observed in cultures inoculated with the strains P36, P102 and P29. The lowest fungal growth was seen in cultures inoculated with PSB strain P104. The two variables AMF hyphal length and bacterial growth along hyphae were not strongly correlated (Pearson correlation coefficient -0.141). Spore production by AMF was also significantly affected by the identity of the PSB strain (ANOVA F _(9, 20)_ = 18.86, *P* ≤ 0.0001). The sporulation response to different PSB strains followed the same trend as for extraradical hyphal growth and these two variables were positively correlated (Pearson correlation coefficient 0.760).

### Experiment 2

**Fig 3:**
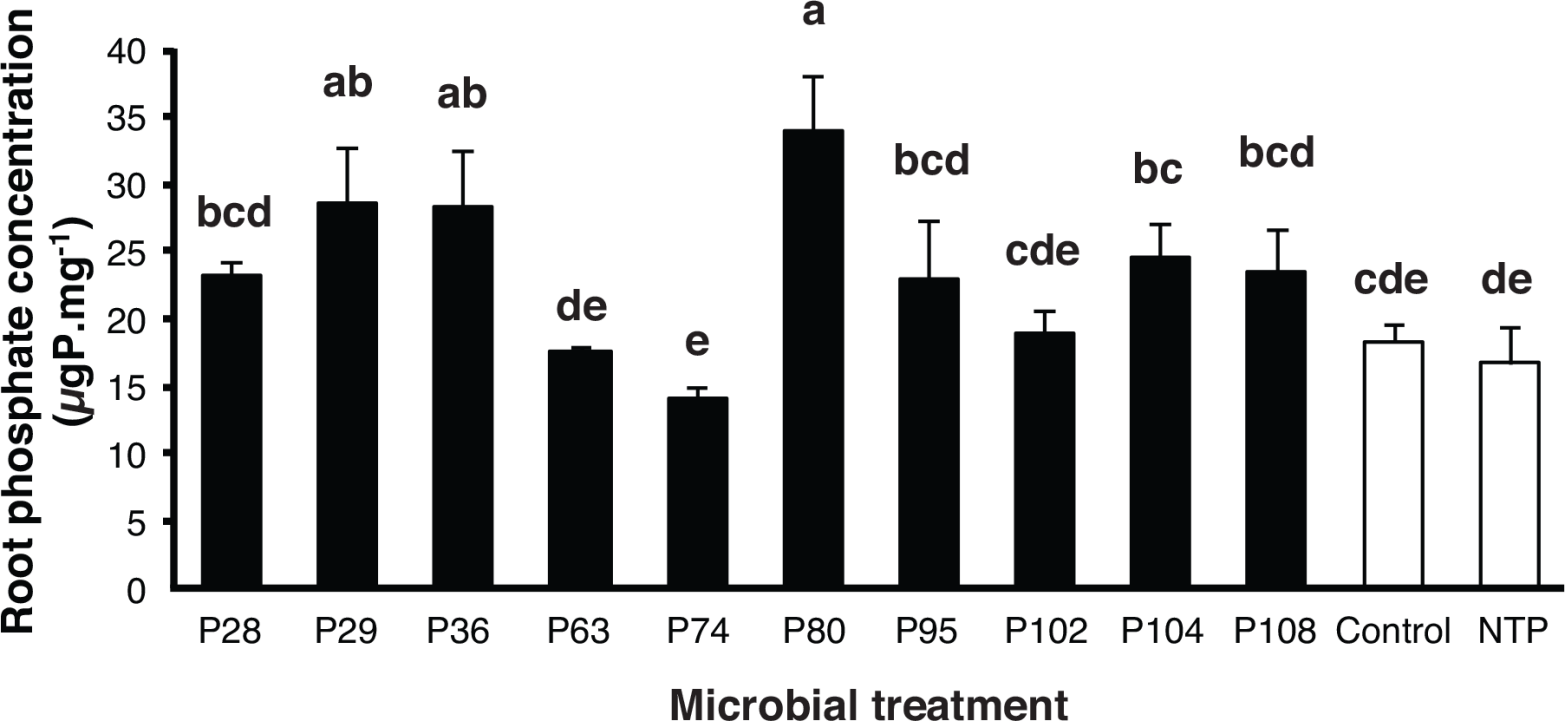
Phosphate concentration in carrot roots colonized by *R*. *irregularis* in experiment 2 and 10 different P solubilizing bacterial strains. The distal compartment contained tri-calcium phosphate. The white bars represent the treatments where roots were colonized by AMF but no bacteria where added, and with tricalcium phosphate (Control) and no tri-calcium phosphate (NTP) in distal compartment. Error bars represent + 1 S.E. Different letters above bars represent significant differences (*P* ≤ 0.05) according to a Tukey test.

In experiment 2 there was no significant difference in P concentration in the carrot roots in the treatment with AMF and lacking bacteria (control treatment) compared to the treatment with AMF but with no bacteria or tri-calcium phosphate (NTP) (Fig 3). This confirmed that AMF hyphae alone were not able to solubilize P and transport it to the roots without bacterial assistance. P concentration in the carrot roots was significantly influenced by which bacterial strain was used to inoculate the distal compartment containing insoluble P (ANOVA, *F*_(11, 24)_ = 6.02, *P* ≤ 0.0001; Fig 3). Three out of ten PSB strains (P29, P36 and P80) significantly enhanced the levels of P in the carrot roots compared to the control or the NTP treatment (Fig 3). The other 7 PSB strains did not significantly enhance levels of P in the carrot roots above that of the control or NTP treatment.

### Experiment 3

At the beginning of experiment 3 the numbers of recoverable rifampicin-resistant mutant bacteria from the surface of tubers was 2.67 x 10^6^ cfu.tuber-seed^-1^ (P95) to 5.63 x 10^7^ cfu.tuber-seed^-1^ (P108). Average numbers of recoverable rifampicin-resistant mutant bacteria from the soil at the end of the experiment ranged from 1.10 x 10^5^ cfu.g soil^-1^ (P36) to 1.40 x 10^6^ cfu.g soil^-1^ (P108). However, due to very high variance among replicates within treatments for these two variables there was no significant difference in the numbers of bacteria per treatment at the beginning of the experiment or at the end of the experiment. Neither of these two variables were strongly correlated with any of the variables of plant growth or fungal colonization measured in Experiment 3. Therefore, the numbers of bacteria that were introduced at the beginning of the experiment and the number recoverable at the end of the experiment cannot explain the results observed on plant P uptake or colonization of the roots by AMF.

**Fig 4:**
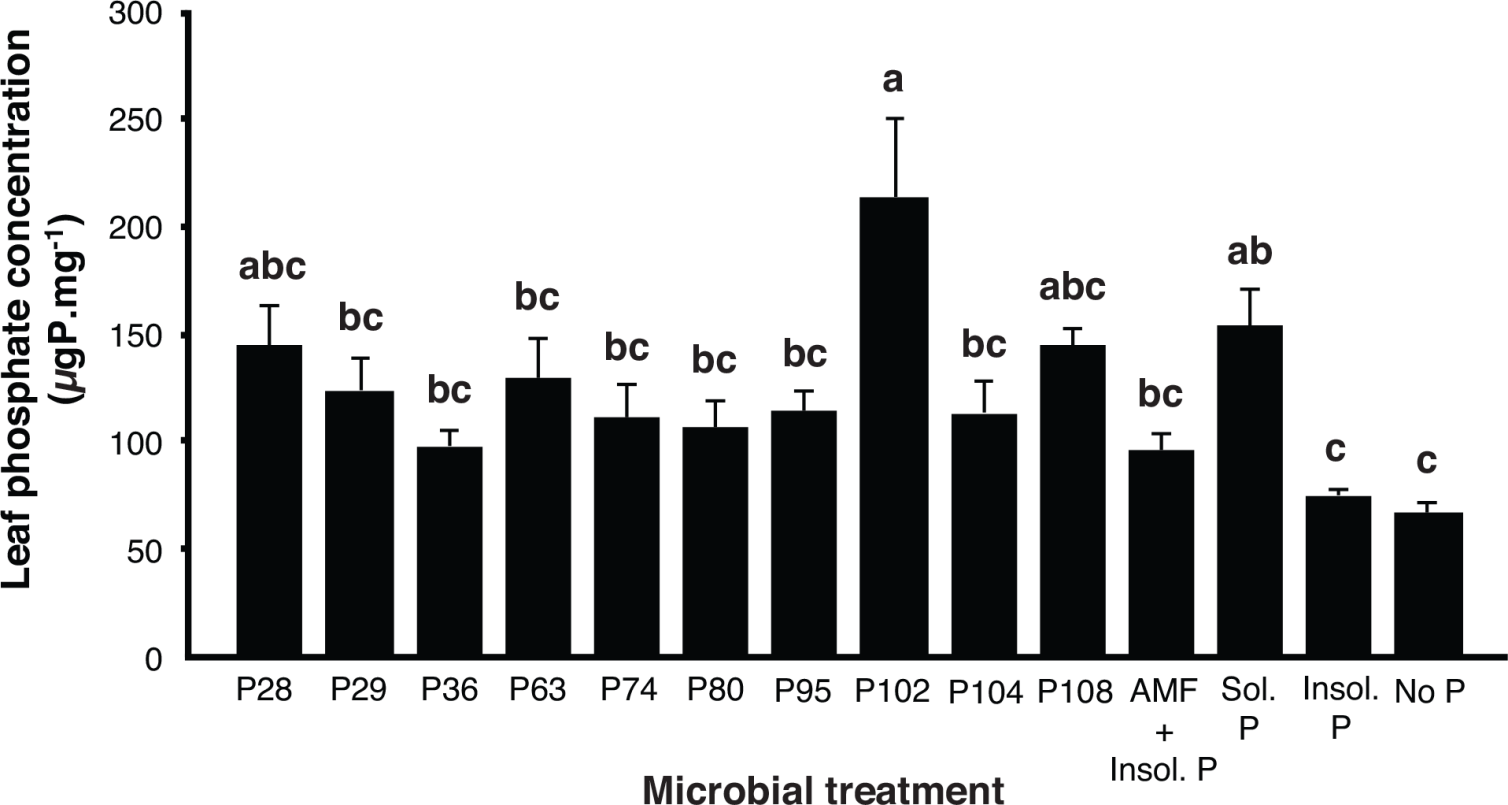
Mean leaf P concentration of potato plants in experiment 3. Plants were inoculated with ten different strains of P solubilizing bacteria (P28 –P108), with added inoculum of *R*. *irregularis* and insoluble P in the form of rock phosphate. AMF + Insol P = added inoculum of *R*. *irregularis* and insoluble P in the form of rock phosphate but no added bacteria. Sol P = addition of soluble P but no added bacteria or *R*. *irregularis*. Insol P = addition of insoluble P in the form of rock phosphate but no added bacteria or *R*. *irregularis*. No P = no addition of any P source and no added bacteria or *R*. *irregularis*. All plants in all treatments are planted in non-sterile soil containing an existing microbial community. Error bars represent + 1 S.E. Different letters above bars represent significant differences (*P* ≤ 0.05) according to a Tukey test.

Leaf P concentration in the plants was significantly affected by the microbial treatments (ANOVA, *F*_(13, 42)_ = 5.53, *P* ≤ 0.0001; Fig 4). The highest leaf P concentrations were found in plants inoculated with AMF and the PSB P102 and in the control where only soluble P (K_2_HPO_4_) was added. Leaf concentration in plants inoculated with P102 was higher than that in 7 of the other PSB treatments, the treatment with AMF and no bacteria and the treatments with rock phosphate and no P that received no additional inoculation with microbes. Leaf P concentration was also significantly higher in the treatment with both AMF and soluble P (Sol. P treatment; Fig 4) than the two controls with rock phosphate and no P that received no additional inoculation with microbes (Insol. P and No P treatments; Fig 4). The leaf P concentration in plants inoculated with AMF, but no inoculation with PSB (AMF + Insol. P treatment; Fig 4), was not significantly higher than the controls (Insol. P and No P treatments; Fig 4) indicating that AMF did not have the ability to assist plant P uptake in the presence of rock phosphate as the only P source.

**Fig 5:**
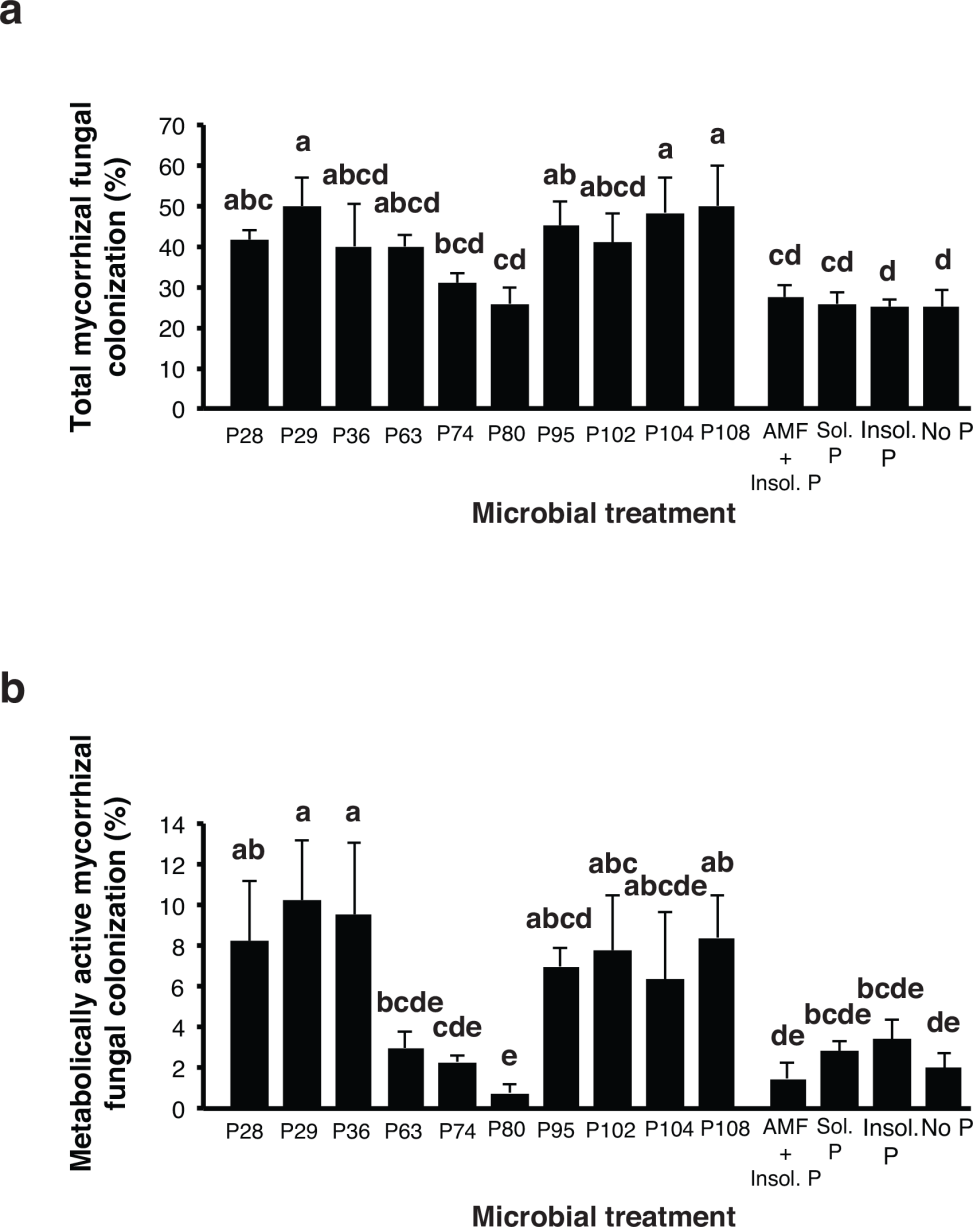
Mean percentage of potato root length colonized by arbuscular mycorrhizal in experiment 3. a. Colonization by all arbuscular mycorrhizal fungal structures. b. Colonization by metabolically active arbuscular mycorrhizal fungal structures, as measured by alkaline phosphatase staining. Treatments shown in the horizontal axis follow those described in Fig 4. All plants in all treatments are planted in non-sterile soil containing an existing microbial community. Error bars represent + 1 S.E. Different letters above bars represent significant differences (*P* ≤ 0.05) according to a Tukey test.

Inoculation with different PSB had a strong and significant effect on mycorrhizal colonization (ANOVA, *F*_(13, 42)_ = 3.12, *P* ≤ 0.002; Fig 5A). Mycorrhizal colonization, as measured by the percentage root length colonised by the fungus, was significantly enhanced in the treatments inoculated with P29, P95, P104 and P108 compared to the treatment inoculated with AMF but without added PSB (Fig 5A). Not all bacterial treatments enhanced mycorrhizal fungal colonization and inoculation with AMF did not significantly increase mycorrhizal fungal colonization over the unsterilized soil that contained a native AMF community. Metabolically active mycorrhizal colonization, measured as percentage root length colonized by mycorrhizal fungal structures stained for phosphatase activity, was also strongly and significantly enhanced in treatments with some PSB strains (ANOVA, *F*_(13, 42)_ = 2.67, *P* ≤ 0.008; Fig 5B). Plants inoculated with P28, P29, P36, P102 and P108 all showed significantly enhanced levels of metabolically active fungal colonisation in the roots compared with the treatment where AMF were added but no PSB were added.

## Discussion

Both AMF and PSBs are common in most soils. Densities of extraradical AMF hyphae in the soil are high and so there is considerable opportunity for PSBs to encounter AMF hyphae. There has been much speculation that PSBs and AMF work synergistically in providing benefit to the plant. For this to happen, these two groups of microorganisms should either interact positively with each other, or at least not be antagonistic with each other. The interactions between these two groups, therefore, needs to be established. The results of this study demonstrated that there was a very wide variation among the 10 *Pseudomonas* strains, that have been shown to have the capacity to solubilize P, in terms of their abilities to grow on the surface of extraradical hyphae of AMF. That the bacteria used in this study strongly influence the growth of AMF, both inside and outside the roots, and in both sterile conditions and in the presence of a native microbial community in the soil is a novel finding. Again such effects on the fungi are highly variable among the different strains. Our results indicate that, depending on the identity of the bacteria, there is a potential for positive interactions between these two microbial groups.

### Fungal effects on PSB growth

The finding that PSBs grow along AMF hyphae is novel. It was already known that bacteria adhere to the surface of AMF hyphae [2, 21] but the bacteria that adhered were never shown to be P solubilizers. *In vitro* experiments also show that several different bacterial groups grow around hyphae of *R*. *irregularis* and that they likely obtain their nutrition from exudates of AMF hyphae [46]. However, no test was made in that study regarding the capacity of the bacteria to solubilize P. All bacterial strains we tested and that had P solubilizing capacity were able to grow on extraradical AMF hyphae. However, there was significant variation among strains in how much the strains could grow on AMF hyphae. We have only used one AMF species in this study and the ability of different PSB strains to grow on AMF hyphae could also potentially be AMF-species specific as variable bacterial communities have been shown to colonize spore surfaces of different AMF species [47]. The observed growth of some PSBs on AMF hyphae could be beneficial for the bacteria in two ways. They could use the hyphae as a route to access further areas of the soil, which could be beneficial for the fungus as P solubilizers could grow away from the route along AMF hyphae into patches containing insoluble P. The bacteria could also use the AMF hyphae as a route allowing growth in the other direction towards the plant and colonize the rhizosphere; an area that could be rich in resources from plant exudates.

Our results also show that measurements of the ability of the bacterial strains to adhere to non-living surfaces are not a predictor of whether the bacterial will be able to adhere and grow on the surface of AMF hyphae.

### Bacterial effects on AMF growth

We have observed two types of differential effects of PSBs on the growth of AMF; namely effects on hyphae and spore production outside the roots in a sterile environment and also colonization by the fungi inside the roots in the presence of a microbial community.

In sterile *in vitro* conditions, the bacterial strains that had the strongest positive effect on the growth of fungal hyphae were not the strains that grew the most on the hyphae (Fig 2). In addition, there was no obvious pattern indicating that the amount of bacterial growth on hyphae or bacterial effects on the growth of hyphae *in vitro* were likely explanations of enhanced P uptake by carrot roots in the presence of certain bacterial strains that were observed in experiment 2 (Figs 2 and 3).

Due to the design of experiment 1, we can conclude that bacteria with ability to solubilize P had strongly differential effects on the growth of AMF hyphae. However, it is not possible to say whether the effects were positive or negative compared to growth in the absence of PSB as hyphal lengths were only measured in the treatments in which PSB were added and not in the bacteria-free control. Therefore, we can only conclude that the different bacteria have positive or negative effects on the growth of AMF hyphae relative to other PSB strains. However, this is an ecologically relevant situation, as AMF probably never grow in the absence of *Pseudomonas* spp. in nature.

In experiment 3, in the presence of a microbial community, AMF colonization inside the roots was also strongly and differentially influenced by the different bacterial strains (Fig 5). In this case, metabolically active AMF colonization, as indicated by alkaline phosphatase staining, was also strongly influenced by the identity of the PSB strain. The percentage of metabolically active AMF colonization of roots was several-fold different in roots inoculated with different PSB strains indicating a very strong differential effect of the bacteria on AMF. Unlike experiment 1, in experiment 3 we can conclude that some PSB strains such as P29, P95, P104 and P108 significantly enhanced AMF colonization over treatments where no PSBs were added. This is a particularly interesting result because in this case the strong effect was observed in the presence of a native microbial community. Whether this effect was a direct effect of the PSB strains on the growth of the fungus or via indirect effects on the microbial community cannot be known. However, a direct effect is certainly possible given the differential effects of the PSB strains on AMF observed *in vitro*.

One important result of experiment 3 was that the soil used in this experiment contained a natural microbial community, including AMF. All treatments therefore contained AMF, including those that were not inoculated with the AMF *R*. *irregularis*. In experiment 3, AMF colonization of roots in control treatments without added *R*. *irregularis* or bacteria was the same as the treatment without added bacteria but with added AMF. This indicates that the AMF inoculation treatment may not have had any effect on mycorrhizal colonisation. Consequently, the differential effects of the PSB strains on AMF colonization must be interpreted as either a response of the native AMF community, or a response of *R*. *irregularis* or the response of both the native community and *R*. *irregularis* together.

### Joint bacterial and AMF effects on P acquisition

The experiments here were not designed to test whether there is a synergistic effect of AMF and PSBs but to assess the influence of one group of microorganisms on the other. Despite this, we can make some additional conclusions from the results of experiments 2 and 3 about how these microbes affect P acquisition.

In experiment 2, carrot roots were not able to obtain any additional P from the insoluble source in the absence of PSB strains. While some PSB strains show P solubilizing activity in standard assays (S1 Table), this did not necessarily lead to enhanced P acquisition by the roots. In fact, standard assays to measure bacterial ability to solubilize P are not a predictor of which strains result in improved P acquisition by roots in this system. In experiment 2, there was no non-AMF control. However, since the bacteria were inoculated, and only subsequently observed in the hyphal compartment, AMF hyphae are an obvious route for any P that was solubilized by the bacteria to have reached the roots. The nature of such a split petri plate culture system would not allow for the rapid movement of solubilized P from the hyphal to the roots compartment. Thus, we conclude that the most parsimonious explanation for the enhanced acquisition of P with some PSB strains was via AMF hyphae.

In experiment 3, while the PSB strains clearly had a very strong effect on the colonization of roots by AMF, this did not translate into obvious patterns of increased P acquisition by the potato plants. One PSB strain enhanced plant leaf P concentration but this bacterial strain did not have a stronger effect on AMF colonization than some of the other strains and overall no microbial treatment resulted in significantly greater plant growth. Moreover, there is a long-standing debate about the suitability for *in vitro* methods, such as tri-calcium phosphate, to predict the microorganisms phosphate solubilizing activity in real soil conditions. Nevertheless, tri-calcium phosphate solubilization, the method used here is the most widely used method to characterise the P solubilization potential of bacteria in *in vitro* conditions and a number of studies have indicated that there is a link between P solubilization activity *in vitro* production of organic acids (e.g. gluconic acid), phosphatases or phytases and actual plant growth promoting activities [7, 48–50]. To demonstrate P solubilization *in situ* by soil microorganisms, is highly complex and not the topic of this article. Certainly, the PSB research community must approach the debate about the suitability of the existent methods to characterise P solubilization abilities, as well as the finding of the most suitable one.

## Conclusions

We conclude that P solubilizing-capable *Pseudomonas* bacteria and AMF can have positive effects on each others growth that could potentially lead to synergism between some combinations. It is significant that some of these strong growth effects were even seen in the presence of a soil microbial community; a test which is often omitted in such investigations. The fact that such enormous variation exists among these bacterial strains in their growth on AMF, and in turn their effects on AMF growth and their influence on P acquisition by roots indicates that effectively using such microbes jointly for improvement of P acquisition by plants needs to consider this variation. On the basis of this investigation, further studies are certainly warranted to investigate possible synergistic effects between members of these two diverse microbial inhabitants of the rhizosphere.

## Supporting Information

S1 Fig Phylogenetic relationships among 63 *Pseudomonas* reference strains and the 10 strains used in this study and isolated from Andean soils. Strains used in this study shown in green. The Maximum Likelihood tree was inferred from concatenated sequences of two housekeeping genes *rpoD* and *gyrB* (1,285 bp). Only bootstrap values greater than 50 are shown. Scale bar 0.02 substitutions per site.
(PDF)

S1 Table Characterization of native strains of *Pseudomonas sp*. in terms of their capacity to solubilize P and produce indol acetic acid (IAA) and indol-related substances *in vitro*. The statistical analysis was carried out through a one-way analysis of variance (ANOVA) and differences between treatments were determined using the Dunnet’s multiple comparison test.
(DOCX)

S2 Table Differing abilities of the 10 bacterial strains to swim, swarm and attach to abiotic surfaces. F ratios in one-way ANOVA were *F*_(9, 20)_ = 10.21, *P* ≤ 0.0001; *F*_(9, 30)_ = 61.48, *P* ≤ 0.0001; *F*_(9, 30)_ = 118.98, *P* ≤ 0.0001. Table shows means value for each variable and each strain followed by the standard error. Different letters in a column denote significantly different means according to a Tukey test.
(DOCX)

S3 Table Detailed analysis of the soil used in Experiment 3. (DOCX)

S4 Table BLAST results for sequences of 4 genes in ten bacterial strains that had been shown to have phosphate solubilizing ability. (XLS)

## References

[1] Smith SE, Read DJ. The mycorrhizal symbiosis San Diego, USA: Academic Press; 2008.

[2] Toljander JF, Artursson V, Paul LR, Jansson JK, Finlay RD. Attachment of different soil bacteria to arbuscular mycorrhizal fungal extraradical hyphae is determined by hyphal vitality and fungal species. Fems Microbiology Letters. 2006;254(1):34–40. 16451176 10.1111/j.1574-6968.2005.00003.x

[3] Marschner P, Crowley D, Lieberei R. Arbuscular mycorrhizal infection changes the bacterial 16S rDNA community composition in the rhizosphere of maize. Mycorrhiza. 2001;11:297–302. 10.1007/s00572-001-0136-7 24549350

[4] Toljander JF, Lindahl BD, Paul LR, Elfstrand M, Finlay RD. Influence of arbuscular mycorrhizal mycelial exudates on soil bacterial growth and community structure. FEMS Microbial Ecology. 2007;61:295–304. 10.1111/j.1574-6941.2007.00337.x 17535297

[5] Rodriguez H, Fraga R. Phosphate solubilizing bacteria and their role in plant growth promotion. Biotechnology Advances. 1999;17:319–39. 14538133 10.1016/s0734-9750(99)00014-2

[6] Browne P, Rice O, Miller SH, Burke J, Dowling DV, Morrissey JP, et al Superior inorganic phosphate solubilization is linked to phylogeny within the *Pseudomonas fluorescens* complex. Applied Soil Ecology. 2009;43:131–8.

[7] Sharma S, Sayyed R, Mrugesh H, Thivakaran A. Phosphate solubilizing microbes: Sustainable approach for managing phosphorus deficiency in agricultural soils. SpringerPlus. 2013;2:587 10.1186/2193-1801-2-587 25674415 PMC4320215

[8] Barroso CB, Nahas E. The status of soil phosphate fractions and the ability of fungi to dissolve hardly soluble phosphates. Applied Soil Ecology. 2005;29:73–83.

[9] Uribe D, Sanchez-Nieves J, Vanegas J. Role of microbial biofertilizers in the development of a sustainable agriculture in the tropics In: Dion P, editor. Soil biology and agriculture in the tropics (Soil Biology 21). Berlin Heidelberg: Springer-Verlag; 2010 p. 235–50.

[10] Walsh UF, Morrissey JP, O'Gara F. *Pseudomonas* for biocontrol of phytopathogens: from functional genomics to commercial exploitation. Current Opinion in Biotechnology. 2001;12(3):289–95. 11404107 10.1016/s0958-1669(00)00212-3

[11] Frey-Klett P, Chavatte M, Clausse ML, Courrier S, Le Roux C, Raaijmakers J, et al Ectomycorrhizal symbiosis affects functional diversity of rhizosphere fluorescent pseudomonads. New Phytologist. 2005;165(1):317–28. 15720643 10.1111/j.1469-8137.2004.01212.x

[12] Owen D, Williams AP, Griffith GW, Withers PJA. Use of commercial bio-inoculants to increase agricultural production through improved phosphorus acquisition. Applied Soil Ecology. 2015;86:41–54.

[13] Rodriguez A, Sanders IR. The role of community and population ecology in applying mycorrhizal fungi for improved food security. ISME Journal. 2015;9:1053–61. 10.1038/ismej.2014.207 25350159 PMC4409159

[14] Toro M, Azcon R, Barea JM. Improvement of arbuscular mycorrhiza development by inoculation of soil with phosphate-solubilizing rhizobacteria to improve rock phosphate bioavailability (P-32) and nutrient cycling. Appl Environ Microbiol. 1997;63(11):4408–12. 16535730 10.1128/aem.63.11.4408-4412.1997 PMC1389286

[15] Gamalero E, Trotta A, Massa N, Copetta A, Martinotti MG, Berta G. Impact of two fluorescent pseudomonads and an arbuscular mycorrhizal fungus on tomato plant growth, root architecture and P acquisition. Mycorrhiza. 2004;14:185–92. 15197635 10.1007/s00572-003-0256-3

[16] Nazir R, Warmink JA, Boersma H, Van Elsas JD. Mechanisms that promote bacterial fitness in fungal-affected soil microhabitats. FEMS Microbial Ecology. 2009;71:161–85. 10.1111/j.1574-6941.2009.00807.x 20002182

[17] Zhang L, Xu M, Liu Y, Zhang F, Hodge A, Feng G. Carbon and phosphorus exchange may enable cooperation between an arbuscular mycorrhizal fungus and a phosphate-solubilizing bacterium. New Phytologist. 2016 10.1111/nph.13838 27074400

[18] Johansson JF, Paul LR, Finlay RD. Microbial interactions in the mycorrhizosphere and their significance for sustainable agriculture. FEMS Microbial Ecology. 2004;48(1):1–13. 10.1016/j.femsec.2003.11.012 19712426

[19] Artursson V, Finlay RD, Jansson JK. Combined bromodeoxyuridine immunocapture and terminal-restriction fragment length polymorphism analysis highlights differences in the active soil bacterial metagenome due to *Glomus mosseae* inoculation or plant species. Environmental Microbiology. 2005;7(12):1952–66. 16309393 10.1111/j.1462-2920.2005.00868.x

[20] Bianciotto V, Bonfante P. Arbuscular mycorrhizal fungi: a specialised niche for rhizospheric and endocellular bacteria. Antonie Van Leeuwenhoek. 2002;81(1–4):365–71. 12448735 10.1023/a:1020544919072

[21] Scheublin TR, Sanders IR, Keel C, Van der Meer JR. Characterisation of microbial communities colonising the hyphal surfaces of arbuscular mycorrhizal fungi. ISME Journal. 2010;4:752–63. 10.1038/ismej.2010.5 20147983

[22] Collavino MM, Sansberro PA, Mroginski LA, Aguilar OM. Comparison of *in vitro* solubilization activity of diverse phosphate-solubilizing bacteria native to acid soil and their ability to promote *Phaseolus vulgaris* growth. Biology and Fertility of Soils. 2008;46(7):727–38.

[23] Jorquera MA, Hernandez MT, Rengel Z, Marschner P, Mora ML. Isolation of culturable phosphobacteria with both phytate-mineralization and phosphate-solubilization activity from the rhizosphere of plants grown in a volcanic soil. Biology and Fertility of Soils. 2008;44:1025–34.

[24] Naik PR, Sahoo N, Goswami D, Ayyaduraj N, Sakthivel N. Genetic and functional diversity among fluorescent pseudomonads isolated from the rhizosphere of banana. Microbial Ecology. 2008;56:492–504. 10.1007/s00248-008-9368-9 18347847

[25] Meyer JB, Frapolli M, Keel C, Maurhofer M. Pyrroloquinoline quinone biosynthesis gene pqqC, a novel molecular marker for studying the phylogeny and diversity of phosphate-solubilizing pseudomonads. Appl Environ Microbiol. 2011;77(20):7345–54. 10.1128/AEM.05434-11 21856827 PMC3194850

[26] Uribe D, Ortiz E, Portillo M, Bautista G, Ceron J. Diversidad de pseudomonas fluorescentes en cultivos de papa de la region cundiboyacense y su actividad antagonista in vitro sobre Rhizoctonia solani. Revista Colombiana de Biotecnología. 1999;2:50–8.

[27] Ceballos I, Ruiz M, Fernández C, Peña R, Rodríguez A, Sanders IR. The in vitro mass-prodcued model mycorrhizal fungus, Rhizophagus irregularis, significantly increases yields of the globally important food security crop cassava. PLoS ONE. 2013;8(8):e70633 10.1371/journal.pone.0070633 23950975 PMC3737348

[28] Bécard G, Fortin JA. Early events of vesicular arbuscular mycorrhiza formation on Ri T-DNA transformed roots. New Phytologist. 1988;108(2):211–8. 10.1111/j.1469-8137.1988.tb03698.x 33874168

[29] Nautiyal C. An efficient microbiological growth medium for screening phosphate solubilizing microorganisms. Fems Microbiology Letters. 1999;170:265–70. 9919677 10.1111/j.1574-6968.1999.tb13383.x

[30] Glickmann E, Dessaux Y. A critical examination of the specificity of the Salkowski reagent for indolic compounds produced by phytopathogenic bacteria. Appl Environ Microbiol. 1995;61:793–6. 16534942 10.1128/aem.61.2.793-796.1995 PMC1388360

[31] Bennasar A, Mulet M, Lalucat J, García-Valdés E. PseudoMLSA: A database for multigenic sequence analysis of *Pseudomonas* species. BMC Microbiology. 2010;10:118 10.1186/1471-2180-10-118 20409328 PMC2873489

[32] Frapolli M, Defago G, Moenne-Loccoz Y. Multilocus sequence analysis of biocontrol fluorescent *Pseudomonas* spp. producing the antifungal compound 2,4-diacetylphloroglucinol. Environmental Microbiology. 2007;9:1939–55. 17635541 10.1111/j.1462-2920.2007.01310.x

[33] Iwamoto T, Tani K, Nakamura K, Suzuki Y, Kitagawa M, Eguchi M, et al Monitoring impact of *in situ* biostimulation treatment on groundwater bacterial community by DGGE. FEMS Microbial Ecology. 2000;32:129–41. 10.1111/j.1574-6941.2000.tb00707.x 10817866

[34] Thompson J, Gibson T, Plewniak F, Jeanmougin F, Higgins D. The CLUSTAL_X windows interface: Flexible strategies for multiple sequence alignment aided by quality analysis tools. Nucleic Acids Research. 1997;25:4876–82. 9396791 10.1093/nar/25.24.4876 PMC147148

[35] Tamura K, Stecher G, Peterson D, Filipski A, Kumar S-. MEGA6: Molecular Evolutionary Genetics Analysis Version 6.0. Molecular Biology and Evolution. 2013;30:2725–9. 10.1093/molbev/mst197 24132122 PMC3840312

[36] Suárez Z, Caballero J, Venturi V. The new group of non-pathogenic plant-associated nitrogen-fixing *Burkholderia* spp. shares a conserved quorum-sensing system, which is tightly regulated by the RsaL repressor. Microbiology. 2008;154:2048–59. 10.1099/mic.0.2008/017780-0 18599833

[37] O'Tool GA, Pratt LA, Watnick PI, Newman DK, Weaver VB, Kolter R. Genetic approaches to study of biofilms. Methods in Enzymology. 1999;310:91–109. 10547784 10.1016/s0076-6879(99)10008-9

[38] St-Arnaud M, Hamel C, Vimard B, Caron M, Fortin JA. Enhanced hyphal growth and spore production of the arbuscular mycorrhizal fungus *Glomus intraradices* in an *in vitro* system in the absence of host roots. Mycol Res. 1996;100:328–32.

[39] Fortin JA, Becard G, Declerck S, Dalpe Y, St-Arnaud M, Coughlan AP, et al Arbuscular mycorrhiza on root-organ cultures. Canadian Journal of Botany. 2002;80(1):1–20.

[40] Tennant D. A test of a modified line intersect method of estimating root length. Journal of Ecology. 1975;63:995–1001.

[41] Murphy J, Riley JP. A modified single solution method for the determination of phosphate in natural waters. Analytica Chimica Acta. 1962;27:31–6.

[42] Huaman Z, Spooner DM. Reclassification of landrace populations of cultivated potatoes (Solanum sect. Petota). American Journal of Botany. 2002;89(6):947–65. 10.3732/ajb.89.6.947 21665694

[43] Trouvelot A, Kough J, Gianinazzi-Pearson V. Evaluation of VA infection levels in root systems. Research for estimation methods having a functional significance In: Gianinazzi-Pearson V, Gianinazzi S, editors. Physiological and Genetical Aspects of Mycorrhizae. France: INRA Press; 1986 p. 217–21.

[44] Tisserant B, Gianinazzi Pearson V, Gianinazzi S, Gollotte A. *In planta* histochemical staining of fungal alkaline-phosphatase activity for analysis of efficient arbuscular mycorrhizal infections. Mycol Res. 1993;97:245–50.

[45] Bago B, Azcon-Aguilar C, Goulet A, Piche Y. Branched absorbing structures (BAS): A feature of the extraradical mycelium of symbiotic arbuscular mycorrhizal fungi. New Phytologist. 1998;139(2):375–88.

[46] Lecomte J, St-Arnaud M, M. H. Isolation and identification of soil bacteria growing at the expense of arbuscular mycorrhizal fungi. FEMS Microbiology Letters. 2011;317(1):43–51. 10.1111/j.1574-6968.2011.02209.x 21219415

[47] Agnolucci M, Battini F, Cristani C, Giovannetti M. Diverse bacterial communities are recruited on spores of different arbuscular mycorrhizal fungal isolates. Biology and Fertility of Soils. 2015;51:379–89. 10.1007/s00374-014-0989-5

[48] Hariprasad P, Niranjana S. Isolation and characterization of phosphate solubilizing rhizobacteria to improve plant health of tomato. Plant and soil. 2009;316:13–24.

[49] Vyas P, Arvind G. Organic acid production *in vitro* and plant growth promotion in maize under controlled environment by phosphate-solubilizing fluorescent *Pseudomonas*. BMC Microbiology. 2009;9:174 10.1186/1471-2180-9-174 19698133 PMC2738680

[50] Oteino N, Lally R, Kiwanuka S, Lloyd A, Ryan D, Germaine K, et al Plant growth promotion induced by phosphate solubilizing endophytic *Pseudomonas* isolates. Frontiers in Microbiology. 2015;6:745 10.3389/fmicb.2015.00745 26257721 PMC4510416

