# Supplementary material for: Bacteria with Phosphate Solubilizing Capacity Alter Mycorrhizal Fungal Growth Both Inside and Outside the Root and in the Presence of Native Microbial Communities
Source: PLoS One. 2016 Jun 2;11(6):e0154438. doi: 10.1371/journal.pone.0154438 (PMC4890779; doi:10.1371/journal.pone.0154438)

Figure S1. Phylogenetic relationships among 63 Pseudomonas reference strains (in black) and the 10 strains used in this study (in green) and isolated from Andean soils. The Maximum Likelihood tree was inferred from concatenated sequences of two housekeeping genes rpoD and gyrB (1,285 bp). Only bootstrap values greater than 50 are shown. Scale bar 0.02 substitutions per site.

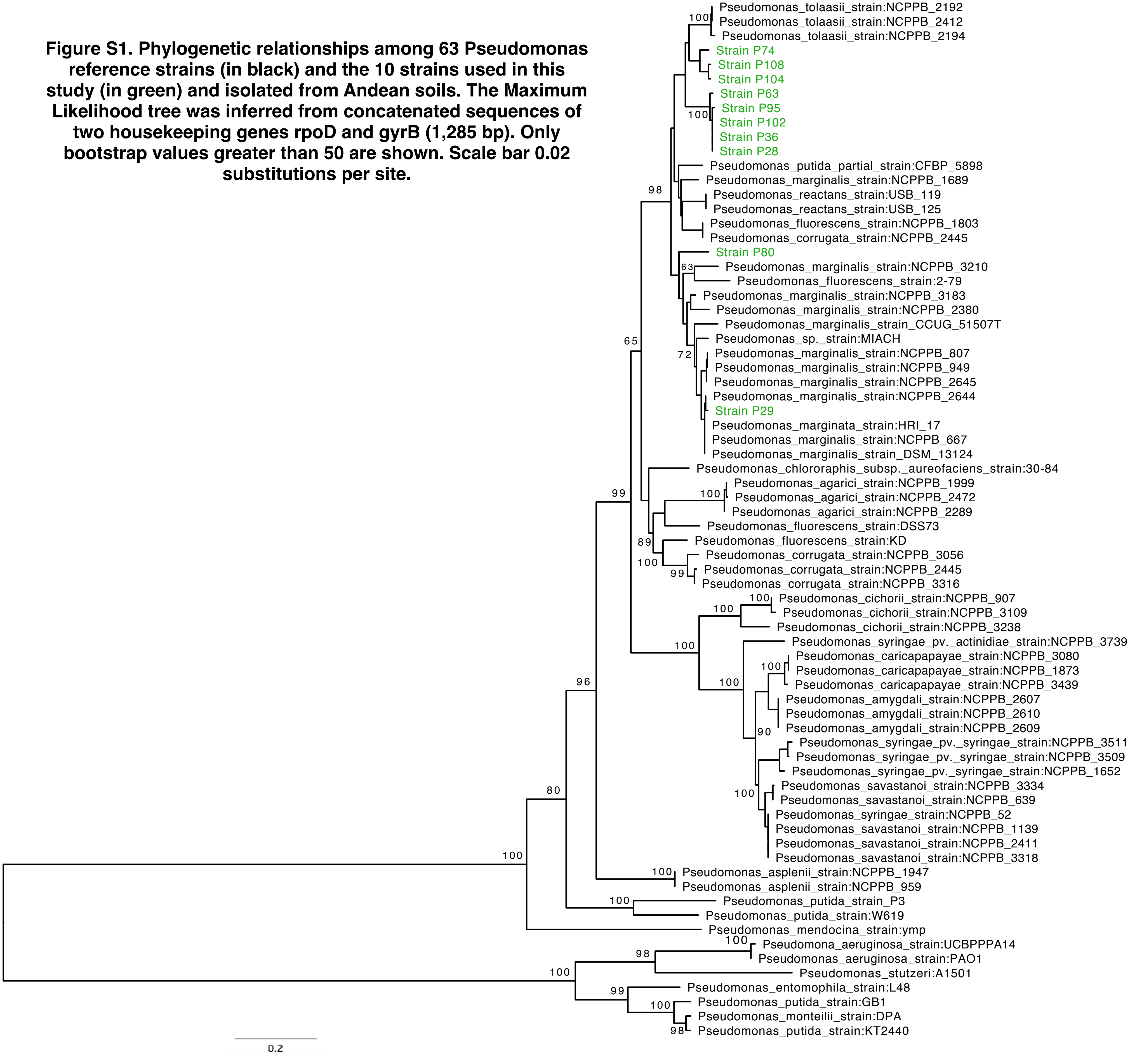

Supplement: S1 Fig — Strains used in this study shown in green. The Maximum Likelihood tree was inferred from concatenated sequences of two housekeeping genes rpoD and gyrB (1,285 bp). Only bootstrap values greater than 50 are shown. Scale bar 0.02 substitutions per site. (PDF) [file pone.0154438.s001.pdf]
