# Supplementary material for: Bacteria with Phosphate Solubilizing Capacity Alter Mycorrhizal Fungal Growth Both Inside and Outside the Root and in the Presence of Native Microbial Communities
Source: PLoS One. 2016 Jun 2;11(6):e0154438. doi: 10.1371/journal.pone.0154438 (PMC4890779; doi:10.1371/journal.pone.0154438)
Supplement: S1 Table — The statistical analysis was carried out through a one-way analysis of variance (ANOVA) and differences between treatments were determined using the Dunnet’s multiple comparison test. (DOCX) [file pone.0154438.s002.docx]

**Supplementary Table 1**. Characterization of native strains of *Pseudomonas sp*. in terms of their capacity to solubilize P and produce indol acetic acid (IAA) and indol related substances *in vitro*. The statistical analysis was carried out through a one-way analysis of variance (ANOVA) and differences between treatments were determined using Dunnet’s multiple comparison test.

| Bacterial strain | Tricalcium phosphate solubilized *in vitro* (mg/L) | Aluminium phosphate  halo | Production of IAA and indol-related substances (μg/ml) | Crop variety/  altitude  (metres above sea level) |
| --- | --- | --- | --- | --- |
| 28 | 102.93 ab | + | 0.593 b | Pastusa/ND |
| 29 | 60.27 b | + | 0 b | Pastusa/ND |
| 36 | 100.75 ab | + | 0.919 b | Pastusa/3050 |
| 63 | 110.28 a | + | 5.267 ab | Pastusa/2631 |
| 74 | 95.55 ab | + | 6.683 ab | Pastusa/2631 |
| 80 | 27.16 c | + | 19.678 a | Criolla/2631 |
| 95 | 114.54 a | + | 2.497 b | Parda/2300 |
| 102 | 101.67 ab | + | 1.395 b | Pastusa/2631 |
| 104 | 46.84 b | + | 11.395 ab | Pastusa/2200 |
| 108 | 33.15 bc | + | 11.307 ab | Parda/2300 |

ND = not determined; + = presence of solubilized halo
