# Supplementary material for: Bacteria with Phosphate Solubilizing Capacity Alter Mycorrhizal Fungal Growth Both Inside and Outside the Root and in the Presence of Native Microbial Communities
Source: PLoS One. 2016 Jun 2;11(6):e0154438. doi: 10.1371/journal.pone.0154438 (PMC4890779; doi:10.1371/journal.pone.0154438)
Supplement: S2 Table — F ratios in one-way ANOVA were F(9, 20) = 10.21, P ≤ 0.0001; F(9, 30) = 61.48, P ≤ 0.0001; F(9, 30) = 118.98, P ≤ 0.0001. Table shows means value for each variable and each strain followed by the standard error. Different letters in a column denote significantly different means according to a Tukey test. (DOCX) [file pone.0154438.s003.docx]

**Supplementary Table 2.** Differing abilities of the 10 bacterial strains to swim, swarm and attach to abiotic surfaces. F ratios in one-way ANOVA were *F*_(9, 20)_ = 10.21, *P* ≤ 0.0001; *F*_(9, 30)_ = 61.48, *P* ≤ 0.0001; *F*_(9, 30)_ = 118.98, *P* ≤ 0.0001. Table shows means value for each variable and each strain followed by the standard error. Different letters in a column denote significantly different means according to a Tukey test.

| **PSB strain** | **Swimming (diameter mm in 24 hours)** | **Swarming (diameter mm in 72 hours)** | **Attachment to abiotic surfaces (OD 600nm)** |
| --- | --- | --- | --- |
| **P28** | 24.67 (SE+) 2.96 ab | 55.00 (SE+) 2.04 b | 2.41 (SE+) 0.10 c |
| **P29** | 25.67 (SE+) 0.67 a | 50.50 (SE+) 6.51 bc | 0.18 (SE+) 0.01 f |
| **P36** | 15.67 (SE+) 1.20 d | 28.00 (SE+) 2.45 f | 4.00 (SE+) 0 a |
| **P63** | 23.67 (SE+) 1.86 ab | 18.25 (SE+) 3.04 f | 4.00 (SE+) 0 a |
| **P74** | 21.00 (SE+) 1.15 bc | 6.75 (SE+) 0.48 g | 2.09 (SE+) 0.08 cd |
| **P80** | 22.33 (SE+) 1.20 ab | 83.13 (SE+) 1.20 a | 0.42 (SE+) 0.06 f |
| **P95** | 18.00 (SE+) 1.15 cd | 81.75 (SE+) 2.93 a | 1.16 (SE+) 0.10 e |
| **P102** | 24.00 (SE+) 1.00 ab | 42.00 (SE+) 6.86 cd | 1.30 (SE+) 0.06 e |
| **P104** | 21.67 (SE+) 1.45 abc | 36.68 (SE+) 1.90 de | 1.79 (SE+) 0.19 d |
| **P108** | 10.67 (SE+) 0.33 e | 6.38 (SE+) 0.31 g | 2.96 (SE+) 0.28 b |
