# Supplementary material for: Bacteria with Phosphate Solubilizing Capacity Alter Mycorrhizal Fungal Growth Both Inside and Outside the Root and in the Presence of Native Microbial Communities
Source: PLoS One. 2016 Jun 2;11(6):e0154438. doi: 10.1371/journal.pone.0154438 (PMC4890779; doi:10.1371/journal.pone.0154438)
Supplement: S3 Table — (DOCX) [file pone.0154438.s004.docx]

Supplementary Table S3. Relevant physical and chemical soil properties of the Colombian Andisol used in Experiment 3.

| Parameter | **Value** |
| --- | --- |
| pH | 6.33 |
| Oxidizable organic carbon (%) | 4.70 |
| Exchangeable Acidity (cmol.kg^-1^) | 1.13 |
| Cation Exchange Capacity (CEC) (cmol.kg^-1^) | 22.98 |
| P: available phosphorus (mg/kg) | 4.70 |
| Soil Texture | Silty |
| Clay (%) | 12 |
| Silt (%) | 58 |
| Sand (%) | 30 |
